# SK Current, Expressed During the Development and Regeneration of Chick Hair Cells, Contributes to the Patterning of Spontaneous Action Potentials

**DOI:** 10.3389/fncel.2021.766264

**Published:** 2022-01-06

**Authors:** Snezana Levic

**Affiliations:** ^1^Center for Neuroscience, University of California, Davis, Davis, CA, United States; ^2^Sensory Neuroscience Research Group, School of Pharmacy and Biomolecular Sciences, University of Brighton, Brighton, United Kingdom; ^3^Brighton and Sussex Medical School, University of Sussex, Brighton, United Kingdom

**Keywords:** SK currents, T-type calcium channels, intracellular calcium, development, regeneration, hair cells, chick, basilar papilla

## Abstract

Chick hair cells display calcium (Ca^2+^)-sensitive spontaneous action potentials during development and regeneration. The role of this activity is unclear but thought to be involved in establishing proper synaptic connections and tonotopic maps, both of which are instrumental to normal hearing. Using an electrophysiological approach, this work investigated the functional expression of Ca^2+^-sensitive potassium [I_K(Ca)_] currents and their role in spontaneous electrical activity in the developing and regenerating hair cells (HCs) in the chick basilar papilla. The main I_K(Ca)_ in developing and regenerating chick HCs is an SK current, based on its sensitivity to apamin. Analysis of the functional expression of SK current showed that most dramatic changes occurred between E8 and E16. Specifically, there is a developmental downregulation of the SK current after E16. The SK current gating was very sensitive to the availability of intracellular Ca^2+^ but showed very little sensitivity to T-type voltage-gated Ca^2+^ channels, which are one of the hallmarks of developing and regenerating hair cells. Additionally, apamin reduced the frequency of spontaneous electrical activity in HCs, suggesting that SK current participates in patterning the spontaneous electrical activity of HCs.

## Introduction

The auditory system of the chicken has been extensively used in comparative studies of the evolution and development of hearing (Carr and Code, 2000). The auditory systems of both humans and chickens are at comparable stages at birth, partially but not fully developed (Jackson and Rubel, 1978). Also, both species start to hear in the second-third of the gestation period (Jackson and Rubel, 1978; Birnholz and Bean, 1983), making the chick cochlea an attractive model system to study the developmental mechanisms of hair cells (HCs). In addition, avian species can regenerate hearing, an ability lost in mammals (recently reviewed in Atkinson et al., 2015).

The avian homolog of the organ of Corti, the basilar papilla (BP), is an auditory epithelium that contains about 10,000 HCs and twice as many supporting cells (Tilney and Tilney, 1986). The HCs are arranged in a tonotopic gradient along the BP, responding to a frequency range of 50–5,000 Hz (Gray and Rubel, 1981; Manley et al., 1996). Low frequencies are detected at the apical end and high frequencies at its base. The two principal morphologically distinct HC types found in the chicken BP are tall and short HCs. The two populations of HCs can be distinguished based on cell size and shape (Hirokawa, 1978; Tanaka and Smith, 1978). Tall hair cells (THCs) are similar to mammalian inner hair cells (IHCs) in location, residing on the superior, or neural, side of the epithelium where the nerve fibers enter the BP. Like the mammalian IHCs, the THCs receive primarily afferent innervation and receive efferent innervation transiently during development (Rebillard and Pujol, 1983; Fischer, 1992). One notable difference between THCs and IHCs is that adult THCs retain some direct efferent contacts in the form of small bouton-like terminals (Takasaka and Smith, 1971; Hirokawa, 1978; Tanaka and Smith, 1978; Fischer, 1992; Zidanic and Fuchs, 1996). Short hair cells (SHCs), on the other hand, are similar to mammalian outer hair cells (OHCs) in location. SHCs are situated on the inferior, or abneural, side of the BP where they are innervated by several efferents that together form a cup-shaped ending (Takasaka and Smith, 1971; Hirokawa, 1978; Tanaka and Smith, 1978; Fischer, 1992; Zidanic and Fuchs, 1996; Ofsie et al., 1997). Outer hair cells exhibit somatic electromotility powered by the membrane-bound protein prestin, which boosts and sharpens cochlear responses sensed by the IHCs (Zheng et al., 2000; Oliver et al., 2001; Santos-Sacchi et al., 2017). However, SHCs exhibit limited motility, suggesting that this may be one of the mammalian specializations enabling high-frequency hearing (He et al., 2003, 2014; Schaechinger and Oliver, 2007; Beurg et al., 2013).

Calcium ions (Ca^2+^) are fundamental to the most important roles in sensory processing mechanisms in vertebrate HCs, including avian and mammalian, such as mechano-transduction, synaptic release, and frequency selectivity (Fettiplace, 2017). Moreover, it is well established that Ca^2+^ ions are instrumental to HCs development, for instance, by supporting the ability to produce spontaneous action potentials, one of the hallmarks of development (Beutner et al., 2001; Marcotti et al., 2003a,b; Jones et al., 2006; Levic et al., 2007, 2011). This developmentally regulated spontaneous electrical activity also reappears in regenerating chick HCs, suggesting that the process of regeneration may partly mimic developmental processes (Levic et al., 2007). This is an important implication to keep in mind when trying to regenerate the sensory epithelium in mammals. Auditory HCs fire spontaneous action potentials before the onset of hearing, which may be a major determinant of synaptic formation and ensuing establishment of proper tonotopic maps along auditory axes during development (Johnson et al., 2011). Once synapses are established between HCs and spiral ganglia neurons, the spontaneous action potentials cease, highlighting the importance of spontaneous action potentials to the development of the auditory inner ear.

In all species examined so far, this spontaneous electrical activity is tonotopically arranged and is developmentally regulated, from apex to base, with the apex firing at lower frequencies and the base at higher frequencies (Levic et al., 2007, 2011; Johnson et al., 2011; Sendin et al., 2014; Jeng et al., 2020). The patterning of the spontaneous action potentials also changes with the development and ceases around P10 in mice and E18 in chicken (reviewed in Levic et al., 2007; Wang and Bergles, 2015).

Spontaneous electrical activity in developing mouse (Marcotti et al., 2003b,^2004^) and chicken (Levic et al., 2007) auditory HCs is a Ca^2+^-dependent transient phenomenon, which also reappears in adult chicken regenerating HCs (Levic et al., 2007). External Ca^2+^, voltage-gated Ca^2+^ currents (VGCC), and intracellular Ca^2+^ availability are important in the initiation and patterning of the spontaneous action potentials (Marcotti et al., 2003b,^2004^; Levic et al., 2007, 2011). T-type VGCC and 4-aminopyridine (4AP) sensitive K^+^ currents were identified as major players supporting this electrical activity in developing and regenerating chick HCs (Levic et al., 2007, 2011). In immature mouse, cochlear HCs action potentials involve L-type, VGCCs, not T-type as seen in chick (Brandt et al., 2003; Johnson et al., 2011; Jeng et al., 2020). The expression of large-conductance Ca^2+^-sensitive potassium (BK) channels coincides with hearing onset and cessation of spike generating ability (Fuchs and Sokolowski, 1990; Kros et al., 1998; Marcotti et al., 2003a). Thus, BK currents do not appear to be involved in the shaping of spontaneous electrical activity in developing HCs.

Small-conductance Ca^2+^-activated K^+^ (SK, K_Ca_2) channels are unique in that they are gated solely by changes in intracellular Ca^2+^(Xia et al., 1998; Adelman et al., 2012); hence, the channels provide a direct link between changes in intracellular Ca^2+^ and membrane potentials. In HCs, the SK channels can be activated by local increases in intracellular Ca^2+^ (Tucker and Fettiplace, 1996; Marcotti et al., 2004) or α9α10 nAChRs (Elgoyhen et al., 2001) activated by the efferent neurotransmitter Ach (Glowatzki and Fuchs, 2000; Oliver et al., 2000). Molecular and electrophysiological data established that the SK2-type channels are present in mature chick SHCs (Yuhas and Fuchs, 1999; Matthews et al., 2005) and mammalian OHCs (Nenov et al., 1996; Dulon et al., 1998; Oliver et al., 2000; Katz et al., 2004; Lioudyno et al., 2004; Marcotti et al., 2004; Nie et al., 2004; Elgoyhen and Katz, 2012). Moreover, the SK2 currents are essential for supporting repetitive Ca^2+^-dependent spontaneous activity in pre-hearing mouse and rat IHCs, which also receive transient efferent innervation (Glowatzki and Fuchs, 2000; Marcotti et al., 2004; Goutman et al., 2005; Johnson et al., 2007, 2013a, b; Kong et al., 2008; Roux et al., 2011; Corns et al., 2018; Frank and Goodrich, 2018; Ceriani et al., 2019). Thus, it is well established that the SK currents have a role in efferent feedback mechanisms and in patterning of spontaneous action potentials in mammalian IHCs (Frolenkov et al., 2000; Kennedy, 2012; Lioudyno et al., 2004; Gómez-Casati et al., 2009; Zorrilla de San Martín et al., 2010; Marcotti, 2012; Fuchs et al., 2014; Katz and Elgoyhen, 2014; Eckrich et al., 2018, 2019; Moglie et al., 2018; Jeng et al., 2020). The presence of the SK currents in developing chick HCs has not been examined so far. Thus, the aim of this article was to investigate the presence and functional contributions of the SK currents in developing and regenerating chick HCs.

## Materials and Methods

### Methods

The methods were very similar to the ones used before (Levic et al., 2007, 2011).

#### Isolation of the Chicken Basilar Papilla

The present investigation was performed in accordance with the guidelines of the Institutional Animal Care and Use Committee of the University of California, Davis. This study includes chickens at different stages of embryonic development ranging from E8 to E21 and post-hatched chickens (P3–P50). Fertilized eggs were incubated at 37°C in a Marsh automatic incubator (Lyon Electric). Chicken embryos were killed and staged according to the number of somites (Hamburger and Hamilton, 1992). Basilar papillae were isolated as described previously (Levic et al., 2007). The preparations were dissected in oxygenated chick saline containing (in mM): 155 NaCl, 6 KCl, 4 CaCl_2_, 2 MgCl_2_, 5 HEPES, and 3 D-glucose, at pH 7.4. The tegmentum vasculosum and the tectorial membrane were removed without any prior enzymatic treatment using a fine needle. Chick basilar papillae were stored in a 37°C incubator in Minimum Essential Medium (Invitrogen, Waltham, MA, United States) before recordings from HCs *in situ* for a maximum of 30 min after dissection. All experiments were performed at room temperature (21–22°C) within 5–45 min of isolation. All the reagents were obtained from Sigma Chemicals (St. Louis, MO, United States).

#### Electrophysiology

Extracellular solution for most experiments contained (in mM): 145 NaCl, 6 KCl, 1 MgCl_2_, 0–2 CaCl_2_, 10 D-glucose, and 10 HEPES, at pH 7.3.

K^+^ currents were recorded in whole-cell voltage-clamp configuration, using 2–4 MΩ-resistance pipettes. Currents were amplified with an Axopatch 200B amplifier (Molecular Devices, San Jose, CA, United States) and filtered at a frequency of 2–5 kHz through a low-pass Bessel filter. The data were digitized at 5–500 kHz using an analog-to-digital converter (Digidata 1200). Action potentials were amplified (100×), filtered (bandpass 2–10 kHz), and digitized at 5–500 kHz using the Digidata 1200 (Molecular Devices, San Jose, California, United States).

The sampling frequency was determined by the protocols used. No online leak current subtraction was made, and as such, only recordings with holding current less than 20 pA were accepted for analyses. The liquid junction potentials were measured (1.3 + 0.6 mV, *n* = 53) and corrected. Capacitative transients were used to estimate the capacitance of the cell, as an indirect measure of the cell size. Membrane capacitance was calculated by dividing the area of the transient by the magnitude of the voltage step. The mean cell capacitance was measured in all experiments (in pF): E8: 5 ± 2 (*n* = 36); E10: 8 ± 2 (*n* = 43); E12: 10 ± 2 (*n* = 122); E16: 12 ± 3 (*n* = 60); E18: 13 ± 2 (*n* = 16); P2: 13 ± 2 (*n* = 10); PT7: 9 ± 2 (*n* = 9); PT15: 11 ± 3 (*n* = 7); PT25: 12 ± 3 (*n* = 5); PT40: 13 ± 2 (*n* = 5). Capacitative decay was fitted with a single exponential to determine the membrane time constant. Series resistance was estimated from the membrane time constant, given its capacitance. This study includes 340 cells with a series resistance (Rs) within the 4–14 MΩ range. After 60–90% compensation, the mean residual, uncompensated Rs was 4.7 ± 0.6 MΩ. The seal resistance was typically 3–10 GΩ.

For whole-cell recordings of currents and action potentials, the pipette solution used contained (in mM): 130 KCl, 10 HEPES, 5 mM D-glucose, 5 KATP, and 0–10 EGTA. Regarding perforated patch experiments, the tips of the pipettes were filled with the internal solution containing (in mM): 150 KCl, 10 HEPES, and 10 D-glucose, at pH 7.3. The pipettes were front-filled with the internal solution and back-filled with the same solution containing 250 μg/ml amphotericin. BAPTA-AM was bath applied to 0 mM EGTA solution.

The stock solutions of all toxins/drugs used were made either in 5% acidic acid (apamin), ethanol (nifedipine, Bay K8644, thapsigargin), or DMSO (kurtoxin) and stored at −20°C. Stock solutions were reconstituted and perfused using a custom-made gravity-fed perfusion system in the recording chamber. The final concentration of the solvents in the recording bath solution was ∼0.001%.

#### Gentamycin Treatment

A single dose (250 mg/kg) of gentamycin was administered subcutaneously to 3-day-old chicks (Bhave et al., 1995; Levic et al., 2007).

#### Data Analysis

The number of cells (*n*) is given with each data set. Data were analyzed using pClamp8 (Axon Instruments, Burlingame, CA, United States), Origin8.6 (Microcal Software, Northampton, MA, United States), and Excel (Excel 2000; Microsoft, Redmond, WA, United States). Pooled data were presented as mean ± SD. Significant differences between groups were tested using ANOVA, *post hoc* Tukey test, with *p* < 0.05 or 0.001, indicating a statistically significant difference.

## Results

### The Small Conductance-Type Ca^2+^-Sensitive K^+^ Current Is Present in Developing Tall Hair Cells and Contributes to the Patterning of Spontaneous Electrical Activity

**FIGURE 1:**
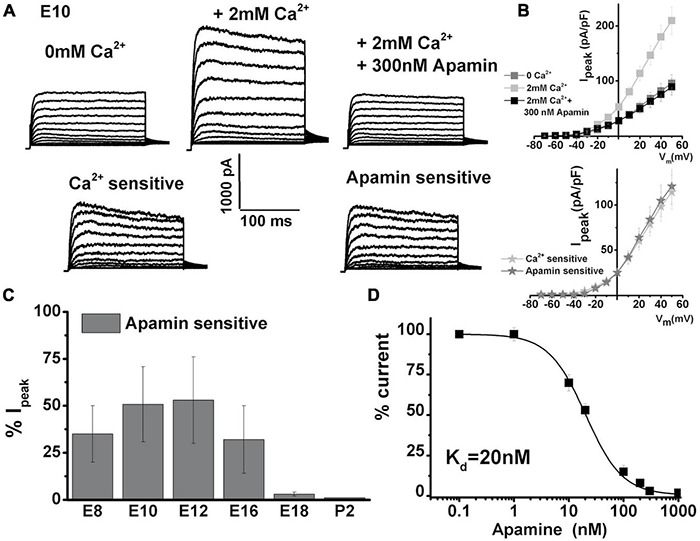
Ca^2+^-sensitive K currents are present in developing chick tall hair cells and are sensitive to apamin. Currents were elicited from midsection neural THCs by 250 ms depolarizing voltage steps in 10-mV increments from a holding potential of –90 mV. **(A)** An example of whole-cell current recorded from an apical THC at E10 without Ca^2+^ added to the bath solution (top left). Increasing the [Ca^2+^]_ext_ concentration to 2 mM produced an immediate increase in the whole-cell current (top middle). The addition of 300 nM apamin abolished most of the effect of [Ca^2+^]_ext_ on the whole-cell current (top right). Ca^2+^-sensitive component (was obtained by subtracting top middle and left) is shown in the bottom left. Apamin-sensitive current (obtained by subtracting top middle and right) looks very similar to Ca^2+^-sensitive currents. **(B)** Plots of the current density–voltage relationships (I–V) for E10 cells, similar to the one shown in panel **(A)**. I–V plot comparing Ca^2+^- and apamin-sensitive components (lower panel) *n* = 15. **(C)** Percentage of total current that is apamin sensitive was measured at +10 mV step potential. Apamin-sensitive current decreased with maturation. An ANOVA was used to show significant differences in the presence of SK current across different developmental ages, *F* (5, 103) = 362.6, *p* < 0.001. The *post hoc* Tukey test showed that there is a significant difference between ages (*p* < 0.001), except between E8 and E16 (*p* = 0.6), E10 and E16 (*p* = 0.6), and E18 and P2 (*p* = 0.9). E8, *n* = 15; E10, *n* = 15; E12, *n* = 23; E16, *n* = 36; E18, *n* = 9; P2, *n* = 10. **(D)** Apamin block of Ca^2+^-sensitive K current was dose-dependent. The half-blocking concentration and Hill coefficient were estimated to be ∼20 ± 6 and 1.3 ± 0.4 nM, respectively, *n* = 10.

To investigate the presence of I_K(Ca)_ in developing longitudinal middle section (midsection) chick, THC whole-cell currents were elicited by 250 ms depolarizing voltage steps in 10-mV increments from a holding potential of −90 mV. Currents were recorded without Ca^2+^ added to the bath solution (Figure 1A, top left panel) and after the addition of 2 mM [Ca^2+^]_ext_, which produced an immediate increase in outward current (Figure 1A, top middle panel). The addition of 300 nM apamin abolished the effect of Ca^2+^ on whole-cell current (Figure 1A, top left panel). Indeed, the profile of I_K(Ca)_ was comparable with apamin-sensitive currents (Figure 1A, bottom panels). The plot of the current–voltage relationship (I–V) for before and after the addition of 2 mM Ca and apamin is shown in the upper panel of Figure 1B, and the I–V plot comparing Ca^2+^ and apamin-sensitive components is shown in the lower panel of Figure 1B (lower panel) for E10 cells (*n* = 15). While the SK currents are exclusively sensitive to intracellular Ca^2+^ availability (Martin and Fuchs, 1992; Kennedy, 2002), increased depolarization could lead to activation of voltage-sensitive potassium currents present in chick HCs (Levic et al., 2011) and accumulation of intracellular Ca^2+^, thereby coupling intracellular Ca^2+^ levels and membrane potential and producing the observed increase in current amplitude (Yamashita et al., 1990).

The average peak current density doubled in the presence of Ca^2+^ [Figure 1B, measured in nominally Ca^2+^-free solution at + 50 mV had an amplitude of 96 ± 16 pA/pF, which increased to 210 ± 24 pA/pF in the presence of 2 mM (Ca^2+^)_ext_]. This current was abolished in the presence of 300 nM apamin (reducing to 89 ± 15 pA/pF). Similar experiments were performed over the developmental range covering the period after HC terminal differentiation (E8) until maturation (P2) (E8, *n* = 15; E10, *n* = 15; E12, *n* = 23; E16, *n* = 36; E18, *n* = 9; P2, *n* = 10). The similarity in Ca^2+^ and apamin-sensitive currents could be generalized in all cells between E8 and E16. The apamin-sensitive component showed developmental upregulation [measured in the% of total current density at + 10 mV, apamin-sensitive component was (in% of total): E7: 32 ± 15; E10: 50 ± 20; E12: 53 ± 21; E16: 42 ± 18; E18:10 ± 3; P2: 2 ± 1]. The currents were compared at + 10 mV step potentials, as this is the peak potential often observed during spontaneous action potentials in developing chick HCs (Levic et al., 2007, 2011). An ANOVA was used to show significant differences in the presence of the SK current across different developmental ages, *F* (5, 103) = 362.6, *p* < 0.001. The *post hoc* Tukey test showed that there is a significant difference between all ages (*p* < 0.001), except between E8 and E16 (*p* = 0.6), E10 and E12 (*p* = 0.6), and E18 and P2 (*p* = 0.9). Lower concentrations of apamin did not produce an effect on whole-cell currents (Figure 1D, *n* = 10). This suggested that of the three identified SK channel isomers, only the SK2 channels are present, as the SK2 channels are sensitive to apamin concentrations in the hundreds of nM range (Figure 1, Marcotti et al., 2004; Stocker, 2004). Together, these results indicate the developmental upregulation of I_K(Ca)_ which is likely to be of the SK2 type in developing chick HCs.

**FIGURE 2:**
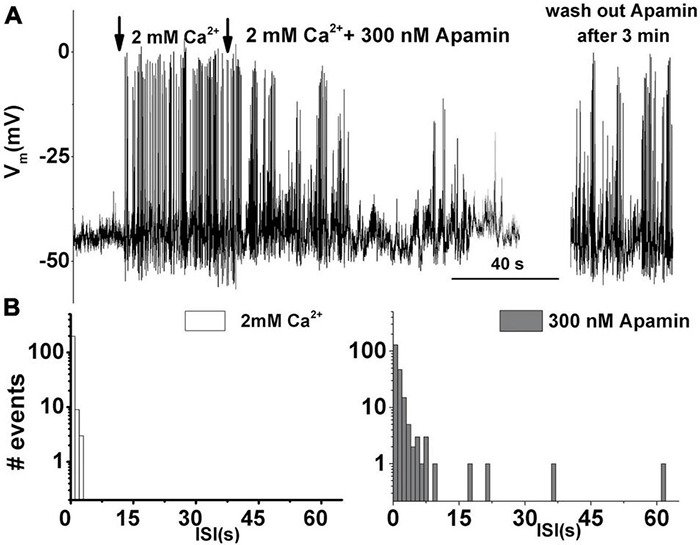
Apamin-sensitive currents contribute to the spontaneous action potential activity in developing tall hair cells. **(A)** An example trace of 200 s continuous recordings of spontaneous activity in the absence and presence of Ca^2+^ and 300 nM apamin. In the absence of [Ca^2+^]_ext_, there is no spontaneous activity. The same cell shows spontaneous action potentials when [Ca^2+^]_ext_ was set to 2 mM. Adding 300 nM apamin abolished this Ca^2+^-dependent electrical activity. Washing out of apamin for about 3 min allowed for the spontaneous action potentials to resume (right). **(B)** The interspike interval distribution histogram of recorded spontaneous action potentials before (left) and after the addition of 300 nM apamin (right). Prolonged interspike intervals suggest that ISK participates in patterning the spontaneous action potentials in developing THCs. Similar data were obtained from other cells: E8 (*n* = 6), E10 (*n* = 10), E12 (*n* = 15), and E16 (*n* = 8).

Next, the role of the ISK in spontaneous electrical activity was examined. As reported previously (Levic et al., 2007), action potentials are driven by Ca^2+^. Spontaneous activity is not possible in the absence of external Ca^2+^ (Figure 2A). However, raising the external Ca^2+^ [(Ca^2+^)_ext_] to 2 mM initiated spontaneous action potentials (Figure 2A). Subsequent perfusion with 300 nM apamin reduced this Ca^2+^-dependent electrical activity (Figure 2A). Washing out of apamin for about 5 min allowed for the spontaneous action potentials to resume (Figure 2A right) indicating that the observed phenomenon is due to SK current block by apamin. The interspike interval (ISI) distribution histogram for spontaneous action potentials before and after the addition of apamin indicates that the ISI time is prolonged with the addition of apamin until eventually all activities cease (Figure 2B). The inhibition of spontaneous action potentials results from diminished repolarization after the upstroke of action potentials, leading to depolarization of cells, which is consistent with observations in developing mammalian IHCs (Marcotti et al., 2004). Together, these results suggested that SK currents determine the patterning of spontaneous action potentials in developing THCs.

### T-Type and L-Type Voltage-Gated Ca^2+^ Currents Differentially Contribute to the SK Current Activation

**FIGURE 3:**
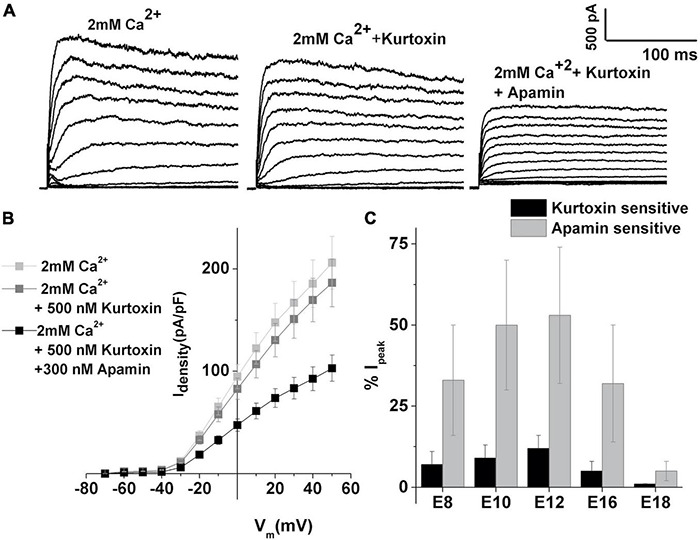
T-type VGCC contributes less to the activation of SK currents. **(A)** Examples of current traces recorded using the protocol as in Figure 1 from midsection THC at E12. Traces are shown before (left) and (middle) after the application of 500 nM kurtoxin and (left) after the application of kurtoxin plus 300 nM apamin. **(B)** Plots of peak current density in 2 mM Ca^2+^, after the addition of apamin, and the subsequent addition of kurtoxin, for the cells shown in panel **(A)**, as in Figure 1. **(C)** Percentage of total current which is kurtoxin or apamin sensitive was measured at +10 mV step potential at E8 (*n* = 15), E10 (*n* = 18), E12 (*n* = 18), E16 (*n* = 16), and E18 (*n* = 7). Kurtoxin-sensitive currents contribute very less to overall apamin-sensitive currents, accounting for about 10% of total calcium-sensitive current.

The expression of SK currents coincides with the upregulation of T-type VGCC in developing HCs (Levic et al., 2007). Thus, any contribution of T-type VGCC on the SK current activation was then assessed. Kurtoxin, a T-type VGCC-specific blocker (Levic et al., 2007), was used to determine the sensitivity of the SK currents to T-type VGCC. The currents were elicited as described in Figure 1 and measured before (Figure 3A left panel) and after the addition of kurtoxin (Figure 3A middle panel), and subsequent addition of apamin (Figure 3A right panel) was used to further block the remaining SK currents. The current density–voltage relationship for this cell is shown in Figure 3B. Measured at +10 mV, currents showed very little sensitivity to kurtoxin compared with apamin (Figure 3C). The kurtoxin-sensitive component of the current also decreased with development (measured at +10 mV,% kurtoxin-sensitive current: E10: 10 ± 3; E12: 12 ± 5; E16: 5 ± 2; E18: 1 ± 1). Thus, T-type currents contribute little to activating the ISK, suggesting the possibility that these channels are loosely coupled and most likely spatially distant.

**FIGURE 4:**
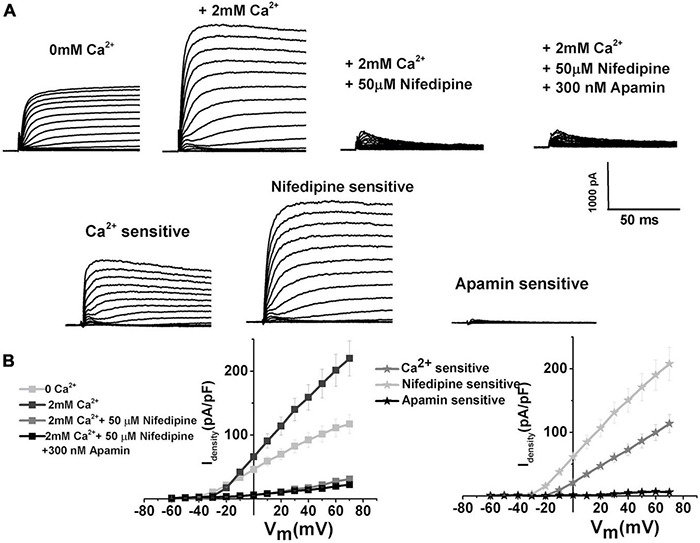
L-type VGCC have a profound effect on the activation of SK currents. **(A)** Examples of current traces recorded using the protocol as in Figure 1 from midsection tall hair cells at E12. Traces are shown before (left), after the application of 2 mM Ca^2+^ (second to the left), after the application of 2 mM Ca^2+^plus 50 μM nifedipine (second to the right), and after the application of Ca^2+^ and nifedipine plus 300 nM apamin (right). **(B)** Plots of peak current densities in 0 Ca^2+^, 2 mM Ca^2+^, after the addition of nifedipine, and the subsequent addition of apamin (right), as well as Ca^2+^, nifedipine, and apamin-sensitive components (left) (*n* = 12). Thus, it is very likely that L-type current is a significant source of intracellular Ca^2+^ leading to ISK activation, even though the nifedipine is a non-specific blocker of L-type VGCC.

While the dominant VGCC in developing chick HCs is of T-type (Levic et al., 2007), the experiments were also conducted to investigate the contributions of L-type VGCC, which are dominant channels in mature chick HCs (Fuchs et al., 1990). Nifedipine, as an established blocker of L-type VGCC in chick (Fuchs et al., 1990; Spassova et al., 2001; Levic and Dulon, 2012) and mouse HCs (Marcotti et al., 2003b), was used to test the contributions of L-type VGCC to SK current activation. The currents were elicited as described in Figure 1 and measured before (Figure 4A left panel) and after the addition of nifedipine (Figure 4A middle panel), and subsequent addition of apamin (Figure 4A right panel) was used to further block any remaining SK currents. While it is evident that nifedipine completely eliminates the apamin-sensitive component, it is worth noting that it also affects the delayed rectifier channels (Valmier et al., 1991; Fagni et al., 1994; Zhang and Gold, 2009; Levic et al., 2011). However, using TEA to eliminate delayed rectifier channels also affected the ISK (data not shown, Lang and Ritchie, 1990; Bond et al., 1999; Blanchet and Dulon, 2001; Dilly et al., 2013). The remaining currents were completely inhibited by 4AP (data not shown, Levic et al., 2011). The average current density–voltage relationship for E12 cells is shown in Figure 4B (*n* = 11). In addition to blocking L-type VGCC, nifedipine (and nimodipine, data not shown) also had non-specific effects on voltage-gated potassium currents. Thus, using these pharmacological manipulations did not help in determining the contribution of L-type VGCC on the activation of ISK. However, the results allow the conclusion that the L-type VGCC has a profound effect on the activation of ISK current. The same effect was observed across all developmental stages.

**FIGURE 5:**
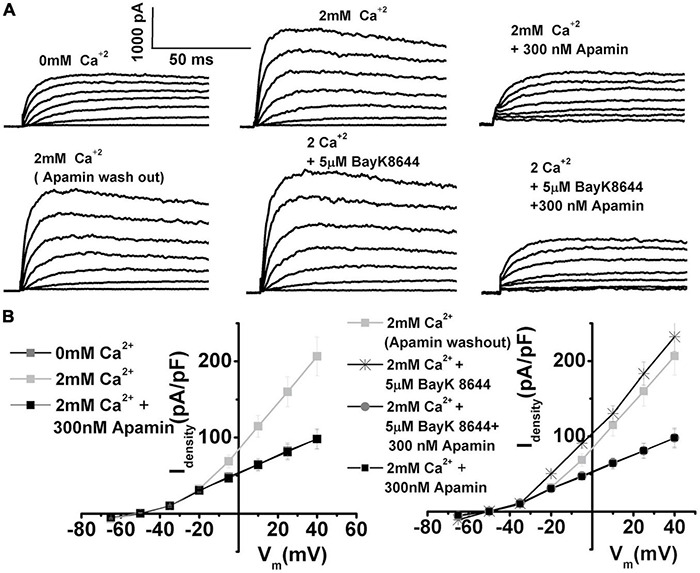
Using Bay K8644, the agonist of L-type VGCC provides further evidence that L-type VGCC contribute to SK current activation. **(A)** Top row: Examples of current traces recorded using the protocol as in Figure 1 from midsection THCs at E12. Traces are shown before (left), after the application of 2 mM Ca^2+^(middle), after the application of 2 mM Ca^2+^ plus 300 nM apamin (left) to determine the size of the apamin-sensitive current in the cell. Bottom row: The current traces recorded in the same cell after apamin wash out (left trace), after the addition of 5 μM Bay K8644 (middle), and after the application of 5 μM Bay K8644 plus 300 nM apamin (right). **(B)** Left panel shows the plots of peak current densities in 0 Ca^2+^, 2 mM Ca^2+^, and after the addition of apamin; right panel shows the plots of peak current densities in 2 mM Ca^2+^ (after apamin washout), and the subsequent addition of 5 μM Bay K8644, and 5 μM Bay K8644 plus 300 nM apamin (*n* = 5). The apamin-sensitive components were of the same profile after the addition of Bay K8644, implying that the Bay K8644 results in further activation of L-type VGCC translated in the direct increase in ISK, which was sensitive to apamin. These results confirm that L-type currents are important for ISK activation, suggesting that these channels are functionally (and possibly spatially) tightly coupled.

Since nifedipine and nimodipine (not shown) affected not only L-type VGCC but also delayed rectifier potassium currents, further experiments were conducted to isolate the role of L-type VGCC on ISK activation. Bay K8644, an agonist of L-type VGCC (Fuchs et al., 1990), was used to directly test the effects of L-type VGCC on the ISK current activation (Figure 5). These experiments involved first determining the amount of the ISK in an individual cell by applying apamin after stimulating ISK by 2 mM Ca^2+^ (Figure 5A top row). After this test, apamin was washed off, and Bay K8644 was applied to measure any further increase in ISK, which was subsequently confirmed by applying apamin to test if the Bay K8644-elicited current were ISK, based on its sensitivity to apamin block (Figure 5A bottom row). Bay K8644 elicited a further increase of 15% in ISK current amplitude as measured at +10 mV in E12 THCs [mean current density (pA/PF): in 2 mM Ca^2+^ 114.8 ± 9.6, after the application of Bay K8644 130.2 ± 10.4]. The experiments revealed that the current elicited by Bay K8644 was indeed fully sensitive to apamin as illustrated in Figure 5A (bottom row), as well as in current density plots (Figure 5B). Therefore, L-type currents contribute substantially to ISK activation, suggesting that these channels are functionally (and spatially) tightly coupled (Zhang et al., 2018).

### The SK Current Is Sensitive to Intracellular Ca^2+^ Availability

**FIGURE 6:**
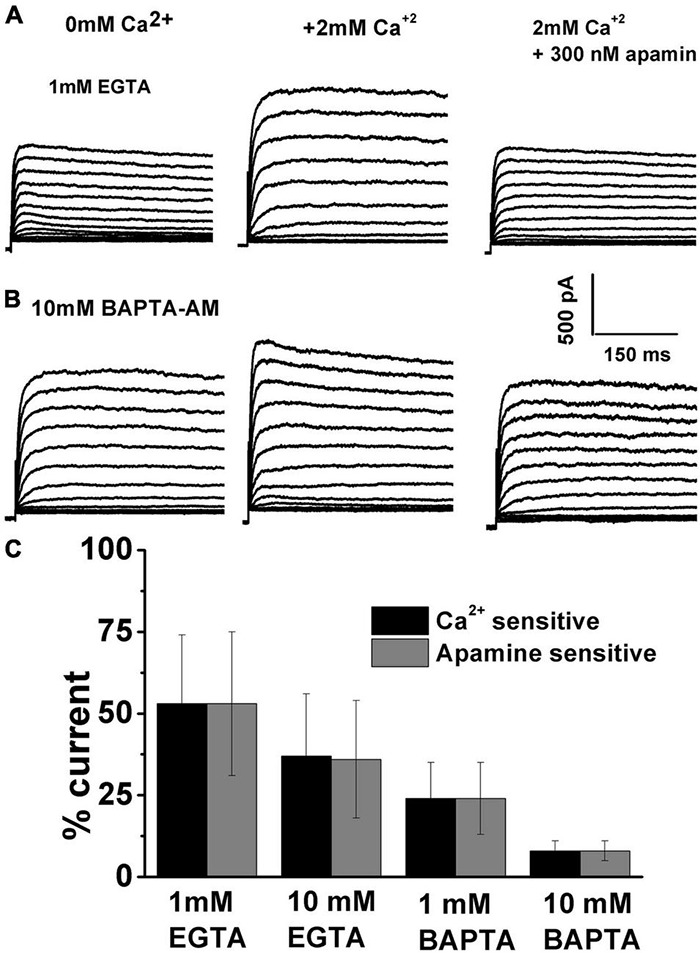
Intracellular calcium affects the SK current activation. Examples of current traces recorded using the protocol as in Figure 1 from midsection THCs at E12. **(A)** Using 1 mM EGTA, recorded traces are shown before **(A)** and **(B)** after the application of 2 mM Ca^2+^, and **(C)** after the application of 2 mM Ca^2+^ plus 300 nM apamin. **(B)** Using 10 mM BAPTA, traces are recorded before **(A)** and **(B)** after the application of 2 mM Ca^2+^, and **(C)** after the application of 2 mM Ca^2+^ plus 300 nM apamin. **(C)** Percentage of total current which is Ca^2+^ and apamin sensitive using 1 mM EGTA (*n* = 11), 10 mM EGTA (*n* = 11), 1 mM BAPTA-AM (*n* = 11), 10 mM BAPTA-AM (*n* = 11). An ANOVA was used to show significant differences in the presence of SK current across different intracellular buffering conditions, *F* (4, 44) = 327.8, *p* < 0.001. The *post hoc* Tukey test showed that there is a significant difference between EGTA and BAPTA buffering conditions (*p* < 0.001), as well as between 1 and 10 mM BAPTA (*p* < 0.001).

The next step was to evaluate the sensitivity of ISK to intracellular Ca^2+^availability (Figure 6). The two well-established Ca^2+^ chelators are BAPTA and EGTA, with BAPTA being ∼150 times faster in binding Ca^2+^ than EGTA (Naraghi and Neher, 1997). Cellular processes that show sensitivity to both BAPTA and EGTA are thought to be within Ca^2+^ microdomains (from ∼50nm to several hundred nm). However, processes sensitive only to BAPTA are localized within “Ca^2+^nanodomains” (within ∼20–50 nm of the Ca^2+^ source) (Naraghi and Neher, 1997; Yu et al., 2014). Thus, a comparison of the effects of BAPTA and EGTA on the activation of SK currents was carried out. Examples of currents recorded before and after Ca^2+^ and subsequently apamin using the intracellular solution containing 1 mM EGTA are shown in Figure 5A and using 10 mM BAPTA-AM are shown in Figure 6B. Ca^2+^ and apamin-sensitive components were similar under all conditions; using BAPTA-AM significantly reduced the ISK (Figure 6C). Measured at + 10 mV at E12, apamin-sensitive components were (% total current): 1 mM EGTA, 53 ± 21; 10 mM EGTA, 36 ± 18; 1 mM BAPTA-AM, 24 ± 11; 10 mM BAPTA-AM, 8 ± 3 (*n* = 11 for each concentration). An ANOVA was used to show significant differences in the presence of SK current across different intracellular buffering conditions, *F* (4, 44) = 327.8, *p* < 0.001. The *post hoc* Tukey test showed that there is a significant difference between EGTA and BAPTA buffering conditions (*p* < 0.001), as well as between 1mM and 10 mM BAPTA (*p* < 0.001). Thus, intracellular BAPTA buffering significantly reduced the SK currents in a concentration-dependent manner (Figure 6).

These findings suggest nanodomain proximity of SK current to the sources of Ca^2+^, creating the possibility that intracellular Ca^2+^ is an important source of the SK current activation. In the cells, a universally important source of intracellular Ca^2+^ is the sarcoplasmic reticulum (SR), where the sarcoendoplasmic reticulum (SR) Ca^2+^ transport ATPase (SERCA) is a pump that transports Ca^2+^ ions from the cytoplasm into the SR.

**FIGURE 7:**
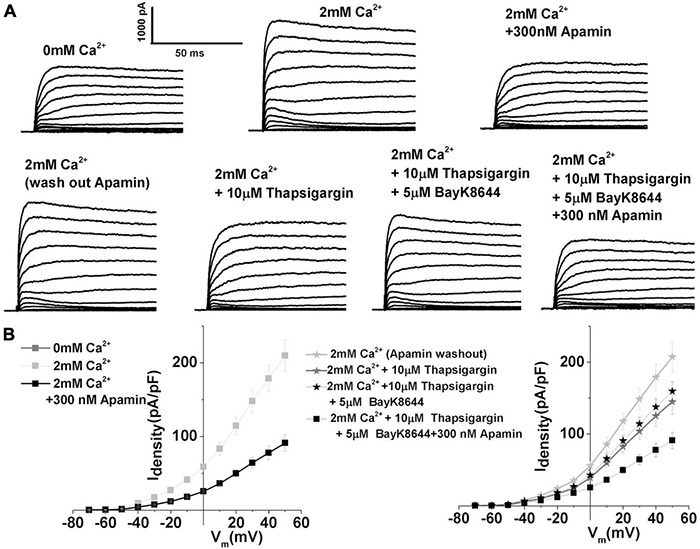
Depletion of sarcoplasmic reticulum calcium stores using prolonged treatment with thapsigargin demonstrates the importance of sarcoplasmic reticulum intracellular Ca^2+^ stores in the activation of SK currents. **(A)** Examples of current traces recorded using the protocol as in Figure 1 from midsection THCsat E12. Top panels: Traces are recorded before (left), after the application of 2 mM Ca^2+^(middle), and after the application of 2 mM Ca^2+^ plus 300 nM apamin (left). This was done to establish the magnitude of ISK in an individual cell. Lower panels show current traces obtained after washing out apamin with extracellular solution containing 2 mM Ca^2+^ (lower left), after 15 min of application of 10 μM thapsigargin in the presence of 2 mM Ca^2+^(lower middle) and after the addition of 10 μM thapsigargin plus 300 nM apamin. **(B)** Right: Plots of the current density–voltage relationships (I–V) for E12 cells, similar to the one shown in panel **(A)** top row. Left: Plots of the current density–voltage relationships (I–V) for E12 cells, similar to the one shown in panel **(A)** bottom row (*n* = 5). ISK current shows sensitivity to thapsigargin, a SERCA pump blocker, suggesting the involvement of sarcoplasmic reticulum Ca^2+^ release in the activation of ISK.

Thus, the sensitivity of ISK to SR Ca^2+^ was probed using thapsigargin, a specific inhibitor of SERCA pumps that leads to intracellular store depletion (e.g., Bian et al., 1991). Prolonged pretreatment with 10 μM thapsigargin (> 20 min) effectively abolished 30% of the total outward current in response to depolarizing voltage step to + 10 mV in E12 THCs (2 mM Ca^2+^ 85.4 ± 8.7, thapsigargin 59.7 ± 5.3). However, the subsequent application of L-type VGCC agonist Bay K8644 increased the magnitude of ISK by 10%, as measured at +10 mV in E12 THCs [mean current density (pA/PF): in the presence of thapsigargin 59.7 ± 5.3; Bay K8644 65.7 ± 7.8] (Figure 5). Thus, these results suggest the importance of intracellular Ca^2+^ availability from SR stores in activating the ISK (Figure 7). The results also demonstrate that Ca^2+^ influx through LTCCs alone is not sufficient to activate SK currents and that SR Ca^2+^ release is important in activating ISK.

### The SK Current Reappears in Regenerating Hair Cells

Spontaneous action potentials initiated by the T-type VGCC are hallmarks of developing and regenerating chick HCs (Levic et al., 2007). The functional changes seen in regeneration seem to share similarity with those seen in development. Thus, the functional presence of SK currents was also assessed in regenerating midsection THCs between 7 and 40 days posttreatment (PT) after inducing HC damage with a single treatment of gentamicin, which causes the loss of HCs, as confirmed with scanning electron microscopy (data not shown, Stone and Rubel, 2000; Levic et al., 2007). During the experiments, it was possible to visually identify the HCs that were newly regenerated, as the cellular appearance markedly differed from the HCs normally seen in the untreated animals. One of the main features was the very immature stereocilia which are starting to form on the apical side. In addition, the cells were tested for the presence of spontaneous action potentials in the whole-cell patch-clamp configuration, which has been identified as one of the markers for regenerating HCs. Also, adjacent cells of similar appearance were tested for the presence of T-type VGCC, another marker for regenerating cells (Levic et al., 2007).

**FIGURE 8:**
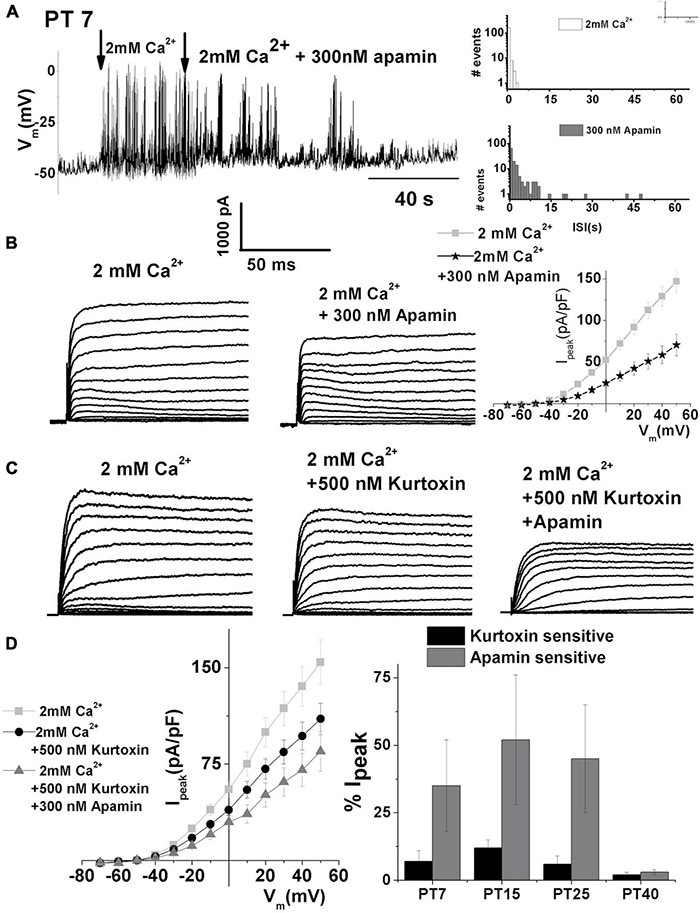
Apamin-sensitive currents contribute to spontaneous action potential activity during the hair cell regeneration. **(A)** An example of continuous recording showing 200 s spontaneous action potentials recorded in PT7 midsection THC in the absence and presence of 2 mM [Ca^2+^]_ext_ after the addition of 300 nM apamin. Right panels show ISI for spontaneous activity in 2 mM Ca^2+^ (top panel) and 300 nM apamin (lower panel). **(B)** Examples of current traces recorded using the protocol as in Figure 1 from midsection THC at PT7. Traces are shown before (left) and after (right) the application of 300 nM apamin. The mean current density–voltage relationship is shown on the right (*n* = 5). **(C)** Examples of current traces recorded using the protocol as in Figure 1 from midsection tall hair cells at PT7. Traces are shown before (left) and after (right) the application of 500 nM kurtoxin, and after 500 nM kurtoxin plus 300 nM apamin. **(D)** The mean current density–voltage relationship for the experiments is shown in panel **(C)** (*n* = 5). Percentage of total current which is kurtoxin and apamin sensitive was measured at +10 mV step potential at PT7, PT15, PT25, and PT40 ISK decreased with maturation in the regenerating tall hair cells. An ANOVA was used to show significant differences in the presence of SK current across different posttreatment days, *F*(4, 22) = 559.3, *p* < 0.001. The *post hoc* Tukey test showed that there is a significant difference between all groups tested (*p* < 0.001): PT7, *n* = 5; PT15, *n* = 7; PT25, *n* = 5; PT40, *n* = 5.

During regeneration, spontaneous electrical activity also showed sensitivity to apamin (Figure 8A, left panel). ISK currents also contribute to the patterning of spontaneous action potentials, as indicated by prolonged ISI (Figure 8A, right). Together, these findings indicate that SK current reappeared again during the process of regeneration. SK currents recorded at PT7 from midsection THCs are shown in Figure 8B. Currents were elicited by 250 ms depolarizing voltage steps in 10-mV increments from a holding potential of −90 mV. Traces are shown before (left) and after (middle) the application of 300 nM apamin. The current density–voltage relationship for the control, apamin, and apamin-sensitive current is shown in Figure 8B. Moreover, since T-type VGCC are also present in regenerating HCs (Levic et al., 2007), their contribution to ISK was determined as shown in Figures 3, 8C. The mean current density plots of these currents are shown in Figure 8D.

Comparing the percentage of apamin- and kurtoxin-sensitive current during the process of regeneration revealed that both kurtoxin-sensitive current and the SK current are transiently upregulated between PT7 and PT25 (Figure 8D right). Measured as the% of total current density at + 10 mV, the apamin-sensitive component was (% of total): (PT7: 27 ± 13; PT15: 53 ± 20; PT25: 42 ± 19; PT40: 3 ± 1). An ANOVA was used to show significant differences in the presence of SK current across different posttreatment days, *F* (4, 21) = 559.3, *p* < 0.001. The *post hoc* Tukey test showed that there is a significant difference between all groups tested (*p* < 0.001). Thus, the SK current transient upregulation is recapitulated during the regeneration of THCs.

## Discussion

Ca^2+^-sensitive K^+^ currents were detected throughout the development of THCs, days before the reported expression of BK channels (Fuchs and Sokolowski, 1990; Li et al., 2009). The identity of the current is most likely type-2 SK current based on its sensitivity to Ca^2+^ and apamin in the nM range (Figure 1). This is in agreement with the molecular, immunolabeling and functional profiles of cloned channels in SHCs (Matthews et al., 2005).

The SK current is developmentally regulated, with the most dramatic expression between E8 and E16, ceasing around E18, which coincides with the reported functional appearance of large Ca^2+^ sensitive K^+^ channels (I_BK_) (Fettiplace and Fuchs, 1999; Li et al., 2009). SK current patterns spontaneous electrical activity in developing and regenerating HCs, where the upregulation of SK current has been observed (Figures 2, 3). SK current regulates spike timing and patterning of spontaneous electrical activity (Figure 2), similar to mouse IHCs (Marcotti et al., 2003a,^2004^), neurons (Llinás, 1988; Baxter and Byrne, 1991), and cardiac myocytes (Xu et al., 2003). Together, these findings strongly suggest that SK current has a pivotal role in the patterning of electrical activity, which may have an instructional role during the development and regeneration of HCs (Figures 2, 8). The past work identified SK current only in short, basal adult HCs in chick, which receive primarily efferent innervation (Yuhas and Fuchs, 1999; Matthews et al., 2005). Thus, the reported developmental upregulation of SK current seems to be a feature conserved across vertebrate species, including turtle, mouse, and rat (Tucker and Fettiplace, 1996; Yuhas and Fuchs, 1999; Marcotti et al., 2004; Matthews et al., 2005; Roux et al., 2011).

During development, THCs transiently receive efferent innervation, which coincides with the observed upregulation of SK current in this study (Rebillard and Pujol, 1983). While the involvement of efferent innervation in the THCs maturation is yet to be established, this efferent innervation is mostly driven by acetylcholine, which was observed in mature chick SHCs receiving primarily efferent innervation (Fuchs and Murrow, 1992; Ohmori, 1993; Hiel et al., 2000), resulting in the increase of K conductance and the rise of the [Ca]_int_ (Ohmori, 1993).

It is well established that Ach released from efferent terminals activates SK current, which transiently hyperpolarizes the HCs (Glowatzki and Fuchs, 2000; Oliver et al., 2000). This hyperpolarization may potentially help in activating the T-type VGCC by removing its inactivation. The current work suggests that the influx of Ca^2+^ via T-type VGCC does not make a substantial contribution to the activation of SK current (Figure 3), implying these channels may be loosely coupled. At the same time, L-type VGCC seems to be a substantial source of intracellular Ca^2+^ for the ISK in chick HCs (Figures 4, 5), which has also been observed in other systems, including mammalian HCs (Marcotti et al., 2004) and cardiomyocytes (Zhang et al., 2018). The availability of intracellular Ca^2^ also affects ISK activation (Figures 6, 7), suggesting the possibility that the SK channels are in close proximity to L-type VGCC as well as other sources of intracellular Ca^2+^, such as SR (Castellano-Muñoz and Ricci, 2014; Castellano-Muñoz et al., 2016).

Furthermore, mouse HCs lacking the SK2 gene also lack spontaneous action potentials and fail to establish proper innervation patterns (Murthy et al., 2009), implicating the importance of SK current in activity-dependent development. Although the role of action potentials in immature IHCs is currently unknown, it has been suggested that they could serve, in analogy with other systems (Moody and Bosma, 2005; Leighton and Lohmann, 2016), as a developmental tool for the functional maturation of the cochlea. In addition, a single action potential is capable of triggering exocytosis of (presumably) neurotransmitters from the HCs in chick (Levic and Dulon, 2012) and mouse (Glowatzki and Fuchs, 2000; Johnson et al., 2007). Thus, this spontaneous activity could be communicated to the brain stem auditory nuclei via afferent nerves. In fact, before the onset of hearing, auditory neurons at various levels of auditory processing show low, bursting, and, sometimes, rhythmic spontaneous activity. This rhythmic activity is robust in the cochlear ganglion cells of the pre-hatched chicks (Jones et al., 2006), and the signal is abolished at higher levels by silencing the cochlea with the tetrodotoxin (TTX) injections or ablation (Lippe, 1994). Therefore, this activity may arise from spontaneous action potentials in developing HCs and could be modulated by SK current.

## Conclusion

This study provides direct evidence that the functional expression of SK current may contribute to the pattering of spontaneous action potentials in developing and regenerating chick THCs. Considering that ISK and T-type VGCC (Levic et al., 2007) are currents identified to reappear during HC regeneration, it would be interesting to see if these currents are necessary and essential components of the regeneration process. If this is the case, modulation of ISK and T-type VGCC currents expression could potentially be utilized for the development of new treatments for damage to or loss of intrinsically non-regenerative mammalian HCs.

## Data Availability Statement

The raw data supporting the conclusions of this article will be made available by the author, without undue reservation.

## Ethics Statement

The animal study was reviewed and approved by the Institutional Animal Care and Use Committee of the University of California, Davis.

## Author Contributions

SL designed the research, performed the experiments, analyzed the data, prepared all the figures, and wrote the manuscript.

## Conflict of Interest

The author declares that the research was conducted in the absence of any commercial or financial relationships that could be construed as a potential conflict of interest.

## Publisher’s Note

All claims expressed in this article are solely those of the authors and do not necessarily represent those of their affiliated organizations, or those of the publisher, the editors and the reviewers. Any product that may be evaluated in this article, or claim that may be made by its manufacturer, is not guaranteed or endorsed by the publisher.
